# Aggressive Metastatic GATA3-Positive Sarcomatoid Carcinoma with Rapid Progression and Invasion of the Liver

**DOI:** 10.1155/2018/6469591

**Published:** 2018-09-10

**Authors:** Irfan Shaukat, Charles Padgett, Maimoona Inayat, Naseruddin Höti, Sadaf Mustafa

**Affiliations:** ^1^Department of Medicine, Med Star Good Samaritan Hospital, Baltimore, MD 21234, USA; ^2^Department of Hematology and Oncology, Med Star Good Samaritan Hospital, Baltimore, MD 21234, USA; ^3^Department of Pathology, Johns Hopkins School of Medicine, Baltimore, MD 21287, USA

## Abstract

*Objective*. GATA3-positive sarcomatoid carcinoma has never been documented in the past. It is a case of aggressive tumor, positive for GATA3, which should be further studied for its prognostic and therapeutic significance.

## 1. Background

Sarcomatoid carcinoma is a biphasic malignant tumor, rarely found in the stomach. This case is about a GATA-binding protein 3- (GATA3-) positive gastric sarcomatoid carcinoma with rapid progression, metastasis, and invasion of the liver leading to death within three months of the diagnosis.

## 2. Case Report

A 50-year-old African-American female with diabetes-mellitus type 2, hypertension, and cardiomyopathy presented with sharp abdominal pain in the right upper quadrant and epigastric region for 4 weeks and an episode of hematemesis with bright red blood and clots.

**Figure 1:**
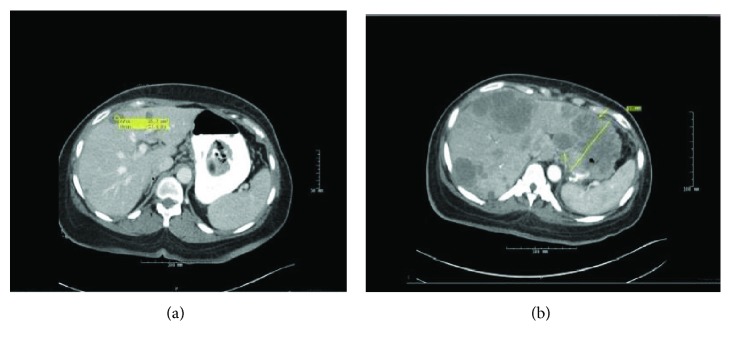
(a) CT imaging of the abdomen and pelvis with IV and oral contrast. Initial presentation with gastric mass and liver metastasis. (b) Repeated imaging on subsequent admission 6 weeks later with an increase in the size of gastric mass and liver invasion and worsening metastasis.

**Figure 2:**
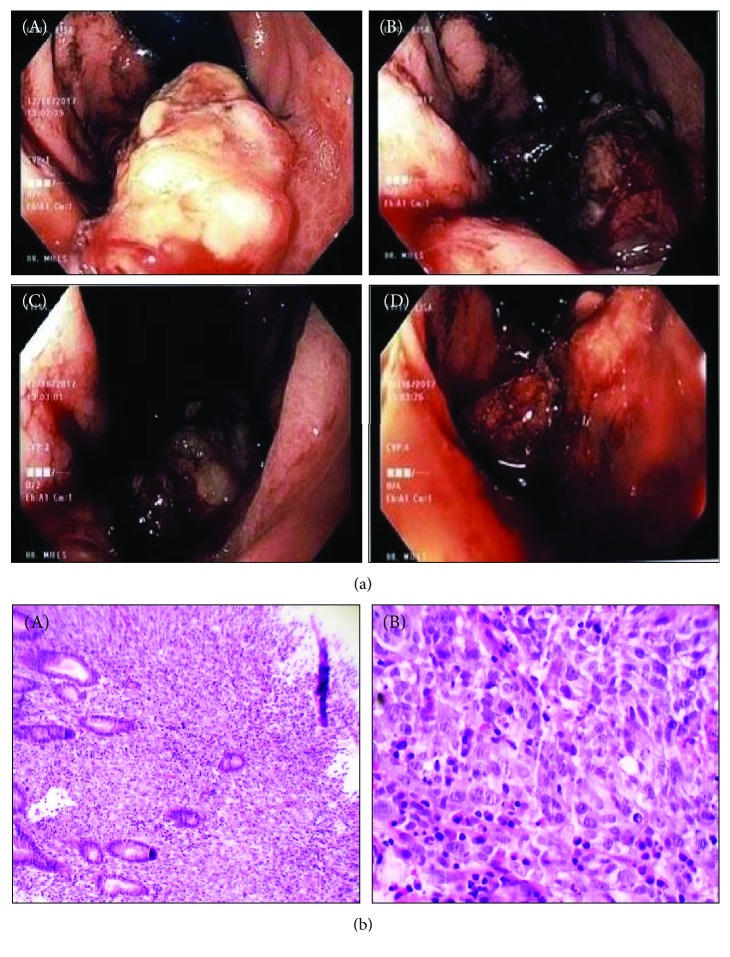
(a) EGD showing lobulated mass measuring at least 3-4 cm in length (A, B) and 2-3 cm in diameter (C, D). (b) H&E staining at lower (4x) and higher magnifications (20x) showing characteristics of gastric carcinoma.

A computed tomography (CT) scan of the abdomen/pelvis with contrast (Figure 1(a)) showed a partially exophytic lobular mass along the superior wall of the gastric fundus measuring 4.5 × 4.3 × 2.0 cm, seven lesions within the liver with the largest measuring 2.2 × 2.0 × 2.5 cm, and a metastatic lymph node anterior to the GE junction. EGD showed a large polypoid fundic mass (Figure 2(a)). H&E staining demonstrated characteristics of gastric carcinoma (Figure 2(b)), with final pathological findings of a sarcomatoid carcinoma which was positive for cytokeratin, SMA, desmin, and GATA3. The patient was discharged to follow up with an oncologist.

She presented again with nausea and vomiting in a couple of months and a repeat CT scan of the abdomen/pelvis (Figure 1(b)) showed an increase in the gastric mass to 8.7 cm with ulceration and invasion of the left lobe of the liver. There was a massive increase in the regional and hepatic metastases with the largest measuring 10 cm, and there was metastasis to the right adrenal gland with associated ascites.

GATA3 is more common in breast or urothelial carcinomas, so a mammogram was performed and reported as BIRADS-1. Also, there was no evidence of any urothelial mass/neoplasm on the earlier CT scans. The patient was transferred to a tertiary care hospital but died before starting therapy.

## 3. Discussion

Sarcomatoid carcinoma is a rare, biphasic, malignant tumor with both epithelial and mesenchymal components, found in the lungs, head and neck, esophagus, thyroid glands, breasts, urinary/genital system, and rarely in the stomach with few cases reported, mostly in the Japanese literature [1, 2].

This case is about an African-American female diagnosed with sarcomatoid carcinoma with EGD showing a polypoid, exophytic, and ulcerated mass like previously reported cases [2]. It was already metastasized at the time of diagnosis and had rapid progression with an increase in size and invasion into the liver, leading to her death within three months of diagnosis. These tumors are mostly diagnosed in the advanced stages with only one case diagnosed as being confined to the mucosa [1]. In a few cases, its components were further differentiated; that is, the sarcomatous component was differentiated into rhabdomyosarcoma, osteosarcoma, leiomyosarcoma, or chondrosarcoma, while the carcinoma component showed neuroendocrine differentiation [2].

The tumor was positive for GATA3, which has never been documented in sarcomatoid tumors of gastric origin. GATA3 is a zinc finger transcription factor expressed in various normal tissues (hematopoietic, mammary gland, skin, inner ear, central nervous system, and kidney) and plays a role in cell differentiation and proliferation [3]. It has mainly been detected in breast and urothelial carcinomas (a sensitive and specific marker for the diagnosis), but it has also been found in neuroblastoma, skin tumors, mesothelioma, ductal carcinomas of salivary glands and pancreas, soft tissue sarcomas, and gastric carcinomas [4].

In studies of breast cancer and gastric adenocarcinoma, GATA3 considered to be a tumor suppressor gene as reduced levels were associated with aggressive tumors and increased metastasis [5]. In contrast, an increased level in soft tissue sarcomas was associated with shorter disease-free and overall survival; thus, an increased level predicted poorer prognosis [6].

GATA3 was detected in 73% of the cases of sarcomatoid urothelial carcinoma in one large study, and thus it is recommended to include GATA3 in the immunohistochemical panel for sarcomatoid carcinomas of unknown origin, especially if a bladder was considered as the primary tumor in the differential diagnosis [3, 7].

Due to the poor efficacy of chemotherapy and radiotherapy in sarcomatoid carcinomas, surgery remains the most effective treatment; however, the prognosis is still poor. In pulmonary sarcomatoid carcinoma (PSC), the efficacy of systemic chemotherapy was reported to vary among patients. In some studies, no benefits on the overall survival (OS) was observed in patients; however, there is a study of tumor response in the Japanese literature for sarcomatoid carcinoma using carboplatin and paclitaxel in combination with bevacizumab [8–11]. While the diagnosis of sarcomatoid carcinoma of the stomach is confined to histomorphological evaluation and immunohistochemistry (IHC), we believe that evaluation for GATA3 in tumor has the potential to predict the prognosis and may function as a useful target for new therapeutic interventions.
